# “I Was Embarrassed to Go and See a Counsellor”: Stigma Experienced by Individuals Diagnosed with Mental Illness (A Systematic Review and Meta-Synthesis)

**DOI:** 10.3390/ijerph23070873

**Published:** 2026-07-04

**Authors:** Oladapo Akinlotan, Dinithi Vidanage, Allen O’connor

**Affiliations:** 1 Faculty of Health, Medicine & Social Care, School of Nursing & Midwifery, Anglia Ruskin University, Bishop Hall Lane, Chelmsford CM1 1SQ, UK; 2 Faculty of Allied Health Sciences, Department of Nursing & Midwifery, General Sir John Kotelawala Defence University, Colombo 10390, Sri Lanka; dinithi@kdu.ac.lk; 3 Faculty of Health, Social Care and Medicine, Edge Hill University, St Helens Road, Ormskirk L39 4QP, UK

**Keywords:** stigma, discrimination, stereotypes, mental illness, systematic review, meta-synthesis

## Abstract

**Highlights:**

**Public health relevance—How does this work relate to a public health issue?**

- Mental health stigma is a major public health issue because it is one of the most pervasive and enduring barriers to recovery, social inclusion and equitable care for people diagnosed with mental illness.
- Although national campaigns and educational initiatives have improved public understanding of mental health, some stigmatising attitudes persist.

**Public health significance—Why is this work of significance to public health?**

- This review offers a contemporary and person-centred understanding of how stigma is experienced by people with mental illness.
- This lived experience of stigma by people diagnosed with mental illness has implications for their everyday functioning and help-seeking behaviour.

**Public health implications—What are the key implications or messages for practitioners, policy makers and/or researchers in public health?**

- Policy making should ensure equitable access to mental health services and protect the rights of mentally ill individuals.
- Continuous research is needed to evaluate interventions and adapt strategies to diverse cultural contexts.

**Abstract:**

**Background:** Stigma is a persistent barrier to psychosocial wellbeing and recovery of individuals with mental illnesses. **Aim:** This study aims to examine stigma experienced by individuals diagnosed with mental illnesses. **Method:** A systematic review and meta-synthesis of peer-reviewed qualitative primary studies followed the Preferred Reporting Items for Systematic Reviews and Meta-Analysis (PRISMA) guidelines. Five databases: CINAHL Ultimate, APA PsycArticles, APA PsycINFO, MEDLINE Ultimate, and Embase were searched for studies published between 2021 and 2025. A total of 17 studies were included after rigorous screening. **Results:** Thematic analysis identified three major themes and fifteen sub-themes. These are manifestations of stigma (prevalence of stigma, operation of stigma, stigma related to mental health diagnosis, stigma related to motherhood and impacts of stigma), multiple factors shaping stigma (ignorance and misunderstanding, spiritual and religious beliefs, family and friends, cultural beliefs) and management of stigma (management strategies, internalising stigma, isolation due to stigma, disclosure of mental illness, non-disclosure of mental illness and help-seeking for mental illness). **Conclusions:** Stigma related to mental illness remains widespread and continues to influence individuals’ experiences, relationships, and help-seeking behaviours. Addressing stigma through improved mental health awareness and supportive environments is essential to promote recovery and wellbeing.

## 1. Introduction

Mental health stigma remains one of the most pervasive and enduring barriers to recovery, social inclusion and equitable care for people diagnosed with mental illness [1,2]. Despite decades of advocacy, policy reform and public education, stigma continues to shape public attitudes, institutional practices and the lived experiences of people who have mental illness [1,3].

Stigma can be “stereotypes”, meaning negative beliefs about the stigmatised group; “prejudice”, described as negative feelings about the stigmatised group; and “discrimination”, signifying behaviour that excludes or marginalises people from the stigmatised group [4]. Stigma can come from within an individual, from others and from the society [2,5]. Self-stigma refers to the internalisation of negative societal stereotypes about mental illness into one’s self concept, reducing hope and self-esteem and undermining recovery [6]. For people with mental health disorders, experiences of stigma are associated with a range of adverse outcomes, including increased symptom severity, feelings of shame, limitations in work and daily activities, reduced help-seeking, and poorer quality of life [5,7,8]. Although national campaigns and educational initiatives have improved public understanding of mental health, some stigmatising attitudes persist, and new forms of stigma continue to emerge [9,10]. Consequently, growing signs of stagnation, and in some cases reversal, in public attitudes highlight the ongoing need to investigate how stigma affects people with mental illness [11].

Given the breadth and complexity of mental health stigma as a concept, several systematic reviews have been conducted in recent years examining different aspects of the phenomenon across populations, conditions and contexts. Habeb et al. [12] conducted a systematic review of mental health stigma over a 30-year timescale, finding that stigma operates across public, self, professional and structural domains and remains a significant barrier to help-seeking, treatment engagement and recovery. Curcio & Corboy’s [7] review looked at stigma specifically in relation to anxiety disorders, and found that stigma is present at public, personal and self-stigma levels, with internalised stigma associated with poorer treatment outcomes and reduced help-seeking. Ferrie et al. [5] looked broadly at mental health but specifically in children and adolescents, finding that stigma is associated with poorer mental health, social rejection, secrecy about difficulties, and reduced help-seeking. A later review into the stigma experiences of young people with anxiety and depression by Ansell et al. [13] found that stigma is emotionally harmful and socially isolating, contributing to shame, concealment of difficulties and reluctance to seek help. Yanos et al. [6] found that internalised stigma lowers self-esteem and leads to poorer social and clinical recovery outcomes among people with severe mental illness. Faleti and Akinlotan [2] examined mental health stigma specifically in Africa.

While previous reviews have examined specific dimensions of stigma, including youth experiences and illness identity, there remains a need for an updated synthesis of qualitative evidence capturing the lived experience of stigma among people diagnosed with mental illness. By focusing on recent in-depth interview studies across diagnostic groups, this review offers a contemporary and person-centred understanding of how stigma is experienced by individuals with mental illness. The aim of this systematic review and meta-synthesis is to explore the lived experience of stigma by individuals diagnosed with mental illness. This outcome will have implications for the everyday functioning and help-seeking of these people. This review is deliberately focused in a way that differentiates it from the existing body of systematic reviews on mental health stigma. While there are a number of reviews in this area, these tend to (a) synthesise evidence across long timeframes [12], (b) focus on particular populations or conditions [5,7,13] or (c) prioritise intervention effectiveness or attitudinal measures [11,14,15]. As a result, there remains a relative absence of a review that captures how stigma is currently experienced, interpreted, and managed by individuals living with mental illness across contexts. This review therefore aims to provide a contemporary, person-centred synthesis that reflects how stigma operates in everyday life including how it is enacted interpersonally, internalised, and navigated through disclosure, concealment, and coping strategies. It is believed that this offers timely insight for both research and practice, particularly in light of emerging evidence that progress in reducing stigma may be stagnating or reversing. This aim will be achieved by restricting the evidence base to the most recent five years (2021–2025), capturing contemporary experiences of stigma in the current social, cultural, and post-pandemic context, including qualitative studies, allowing for an in-depth synthesis of meaning, rather than aggregating prevalence or attitudes, and focusing explicitly on the lived experience of stigma, rather than on public attitudes, interventions, or specific diagnostic or demographic subgroups.

## 2. Methodology

This is a systematic review of peer-reviewed qualitative primary studies that examine stigma experienced by individuals diagnosed with mental illnesses. This review follows the Preferred Reporting Items for Systematic Reviews and Meta-Analysis (PRISMA) guidelines [16], with details in the Supplementary Materials, and was registered with PROSPERO (Reference number: CRD420251245430).

The search was completed on 7 November 2025 using five databases, namely CINAHL Ultimate, APA PsycArticles, APA PsycInfo, MEDLINE Ultimate, and Embase. The following search strings were used on all the databases: (stigma or stigmatization or stigmatisation or shame or discrimination or prejudice) AND (discrimination or prejudice or stereotype or bias or stigma) AND (mental health or mental illness or mental disorder or psychiatric illness). The filters applied were: publication date: 2021–2025; peer reviewed journals; source types: academic journals; age group: adolescents and adults; language: English; and methodology: qualitative study. Qualitative or mixed-methods studies with a distinct qualitative component published between 2021 and 2025 in peer-reviewed academic journals in the English language were considered. The review included adolescents and adults diagnosed with mental illness. Studies were required to report lived experiences of mental health-related stigma.

Studies employing in-depth interviews (individual or face-to-face interviews, telephone or online) as the primary data collection method were included to capture detailed, subjective, and personal accounts of stigma. This approach was selected to prioritise the exploration of personal lived experiences, allowing participants to express individual perceptions, emotions, and meanings in depth [17,18]. Studies using focus group discussions and open-ended questionnaires were excluded. While these methods can provide valuable insights, focus groups may be influenced by group dynamics and social desirability, which can limit the expression of sensitive individual experiences. Similarly, open-ended survey responses may lack depth and opportunities for probing compared to interactive interviews [19,20].

**Figure 1:**
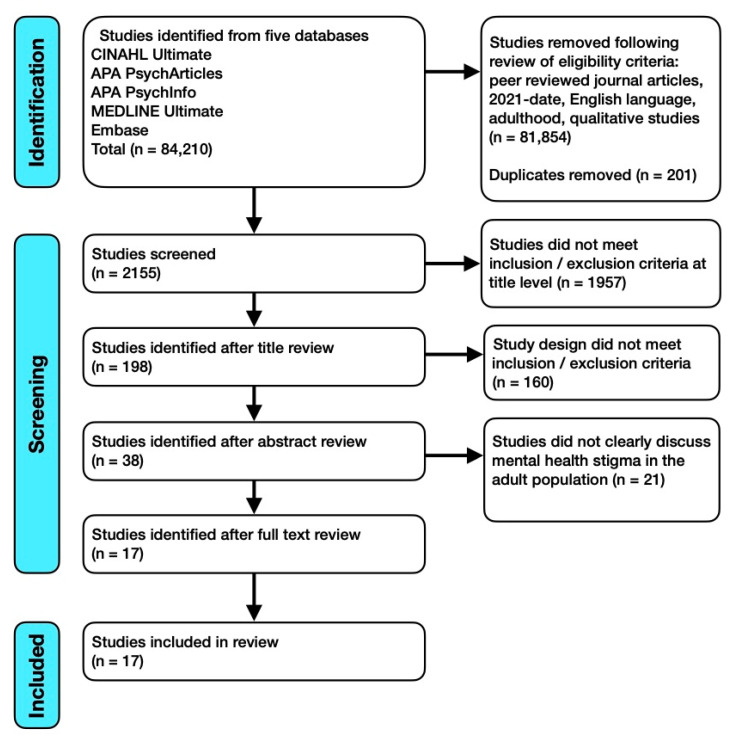
PRISMA flow diagram of the study [16].

In the initial search, a total of 84,210 studies were identified from databases. After filters were applied, 81,854 studies were removed and duplicates of 201 were removed as well. A total of 2155 studies were screened for title/abstract and 1957 were removed. The remaining 198 were screened for pre-defined inclusion/exclusion criteria (Table 1) and 160 studies were removed. Out of the remaining 38 studies, another 21 were removed as they did not discuss the individual experience of stigma related to mental health. As a result, a total number of 17 studies were identified. A review of the reference lists of the 17 identified studies yielded no new relevant studies. The grey literature was not searched as the focus of the review is on peer-reviewed academic journals. Finally, a total of 17 studies were selected for the final review (Figure 1). All three reviewers were involved in the search, screening and selection of studies.

In order to provide a summary of the characteristics of all the selected studies (such as author, year of publication, country, aim, study design, major findings, and limitations) all the included studies were extracted into Microsoft Word (Table 2). The quality of the 17 included studies was assessed using the Critical Appraisal Skills Programme [21]. All the included studies have a minimum score of 85% and are judged to be excellent based on CASP classification [21] (Table 3). On the other hand, the relationship between researcher and participant (reflexivity) is inadequately addressed in all the selected studies and this may affect transferability of findings. ROBIS (Risk of Bias in Systematic Reviews) assessment was conducted to assess the risk of bias for the review [22]. Overall, the review demonstrates a generally low to moderate risk of bias, with the only major concern being the limited reporting of reflexivity in the selected studies (Table 4).

For the data synthesis and meta-synthesis, thematic analysis was used [19]. All text from the ‘Results/Findings’ section of each study was extracted and imported into Microsoft Word. The results/findings of all the included studies were subjected to initial and independent coding by two reviewers. Coding involves providing a summary of each sentence from the results/findings of all the included studies. Initial coding results were then compared among the two reviewers before descriptive coding was carried out by one independent reviewer. Agreements amongst reviewers were reached via email exchange. Following agreement amongst all three reviewers, descriptive codes were then collapsed into emerging themes. This was achieved by identifying patterns among the descriptive codes to identify possible themes, followed by reviewing and refining these likely themes to create concise and easily understandable themes [19]. Themes were identified solely from the descriptive codes and not based on a pre-defined matrix or an existing list of themes. Finally, all the recurring and common themes that emerged from all the included studies were compared and analysed to identify the final major themes and sub-themes. All three reviewers reviewed and agreed on the final themes and sub-themes.

## 3. Results

**Figure 2:**
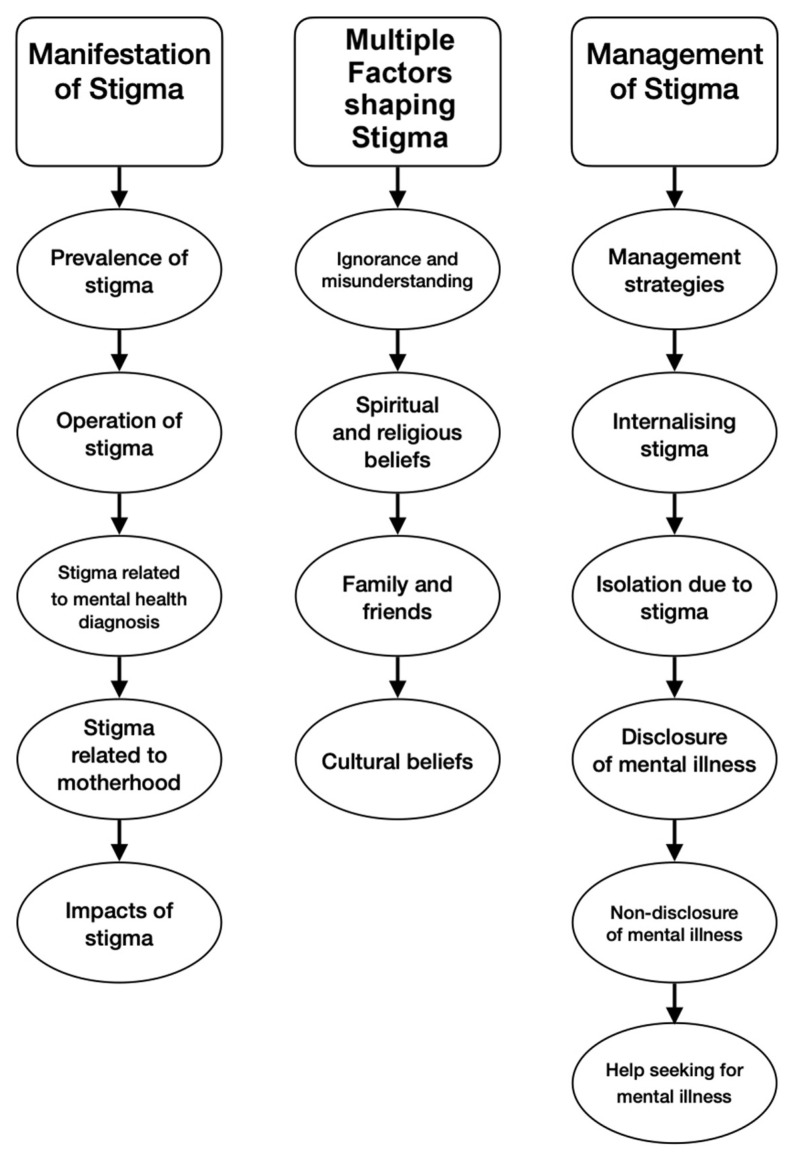
Themes and sub-themes identified from the study.

The thematic analysis and meta-synthesis of all the included studies identified three major themes (manifestation of stigma, multiple factors shaping stigma and management of stigma) and fifteen sub-themes discussed below (Figure 2).

### 3.1. Manifestation of Stigma

The first theme, manifestation of stigma, has five sub-themes, namely prevalence of stigma, operation of stigma, stigma related to mental health diagnosis, stigma related to motherhood and impacts of stigma (Figure 2).

#### 3.1.1. Prevalence of Stigma

Mental illness is universally perceived as a source of stigma [26] which often came from stereotypes such as negative views of others about mental illness [31]. Mental health stigma is pervasive [13] and it is a persistent and underlying influence driving many of the challenges faced by people with mental health illness [28]. The omnipresent nature of stigma means that people with mental illness are frequently exposed to stigmatising attitudes and behaviours across all spheres of life [13,27]. Despite increased public discussion of mental health, awareness alone has not removed prejudice or discrimination, and stigma persists as a social reaction that devalues people with mental illness [13,24].

*“Mental health stigma is a very prevalent issue, even though that I’ve seen some development, uh, but still people try to hide it. And that—that’s very sad….” (Hania, 23, female)*.

[26]

*“There’s a lot of stigmas there, really. And yeah, I think that’s definitely affected me as well” (P06, 22, female)*.

[30]

Media and celebrity coverage have been trying to alter stigma content by reshaping public perceptions of mental illness [24] but they often misrepresent some mental illness thereby creating mixed or negative portrayals [24].

*“The judgment or the stigma that people have on schizophrenia and what they see in movies. Like they think we are the psycho killer or something”*.

[32]

Three major types of stigma were identified: self-stigma, public stigma and professional stigma [23,32,35,36]. Some people felt negative about themselves because they were not “normal” due to their mental illnesses [32] while some people reported experiencing stigma from healthcare professionals [36]. Some families/friends experienced shock, denial, anger and dismay with the public stigma upon the diagnosis of a mental disorder of their loved ones [35].

#### 3.1.2. Operation of Stigma

Stigma operates daily across interpersonal, occupational, and community contexts, shaping lived experiences of mental illness [28]. Stigma most often occurs via direct communication, indirectly through subtle comments, behaviours, indirect messages or wider media narratives [13]. People with mental illness have described their experience of stigma including: dismissing symptoms or questioning legitimacy of illness [26]; accusations of faking or exaggerating mental health problems to gain attention from others or for personal benefit such as avoiding responsibilities [13,33]; negative labels such as ‘crazy’, ‘pagal’ or, “mentally ill person” [26,36], portraying them as clingy, needy, or restless and framing them as difficult companions [13]; describing mental health problems as a trend for young people [13]; describing mental distress as an evaluation of capability in education and work [13]; framing emotion or behaviour as a simple choice shift and implying personal fault [13]; giving advice that trivialises symptoms and suggests effortless solutions [13]; and social exclusion including being discriminated, marginalised, rejected and avoided by families, friends and colleagues [23,26,28,36,37].

*“(They’re told that) it’s not real in a sense—the way that they’re feeling is not real” (Steph, 20, female) and “everyone has sad days but you’re just exaggerating” (Jane, 22, female)*.

[13]

*“I had a psychiatrist say to me…when I couldn’t get out of bed, ‘you just don’t want to go to school’” (Maggie, 19, female)*.

[13]

*“I was told that I was attention-seeking” (Verity, 18, female)*.

[13]

#### 3.1.3. Stigma Related to Mental Health Diagnosis

Young people reported broadly comparable experiences of stigma and its impact regardless of whether they had received a formal diagnosis of depression [30]. For some people, a named mental health diagnosis provided validation, reduced self-esteem, and provided insight and understanding, relief, and greater appreciation of mental health as part of their identity [26,30]. However, for others, receiving a formal diagnosis reinforced persistent feelings of inadequacy and reduced their sense of personal value or usefulness [30].

*“I think the fact that before I was diagnosed, I also thought it was just a bunch of nonsense. I think in the beginning I was quite mad, um, I was dealing with it for such a long time, and I was also just thinking that this is nonsense. You know, when I was diagnosed, I was like, ‘Okay, I’m one of those people now’” (P03, 24, female)*.

[30]

*“It made me feel like I am the problem. I’ve always thought like I’m a problem and having someone else confirm it just made me feel worse because I try so hard, but at other times really, I struggle” (P17, 22, male)*.

[30]

Young people who completed high school reported more stigma compared to those who did not and a higher proportion of individuals who had high school experience reported more negative views of serious mental illness compared to those who had not [32]. Psychosis is one of the most stigmatised mental health problems associated with negative labels such as crazy, violent, a drug addict, and a danger to society [38]. As a result, young people with psychosis continually adapt their behaviour and decisions to manage both anticipated and experienced stigma [31]. This is because stigma worsens their psychotic experiences, including voices and paranoia [38].

#### 3.1.4. Stigma Related to Motherhood

Being a parent with a mental illness brings stigmatising narratives such as ‘mentally ill mother’, ‘bad mother’, ‘crazy women’ and ‘weak women’ but some mothers want to be seen as a capable, strong, responsible, and disciplined person [34]. Mothers with mental illnesses strategically use contrast devices to position their identities against negative labels [34]. Some individuals compare their mental illness to others to help identify a justification to avoid potential blame [34]. People diagnosed with post-traumatic stress disorder (PTSD) were not too worried about their condition compared to those who have schizophrenia because unlike the other diagnoses, PTSD is something that can eventually be resolved [34]. This is based on some narratives that people with schizophrenia are dangerous, people with depression are lazy or weak, or people with borderline personality disorder are manipulative [34].

*“I still, no matter how I feel, go out with them every day or to the park”, and “The children also get everything from me”*.

[34]

*“It makes it a bit easier, when I tell people what I’ve been through, that it’s depression and not psychosis. Because it’s easier for people to understand that you get over depression, than that you get over psychosis” (Isa, 17 years)*.

[27]

#### 3.1.5. Impacts of Stigma

Stigma results in sustained and long-term negative effects on self-worth, family, relationships, social life, work, studies, physical health and overall wellbeing of individuals [30,35]. It leads to shame and embarrassment, self-criticism, low self-esteem, and reduced opportunities, impacts working capacity, undermines trust in previously supportive relationships, causes lack of support by family and friends due to misconceptions of the illness, and causes sadness and emotional distress, all which can influence people’s future decisions [29,31,32,33,37,38].

*“I have very low self-esteem, low self-confidence, self-worth, all of it. I have an inferiority complex. I am trying and learning very gradually but slowly and surely to love myself again” (participant 1)*.

[38]

*“The fear of stigma because, if I get anxious, the voices get worse. So its kind of a vicious circle, I need to go out to get more confidence in these sorts of things. Then I get more anxious and then the voices get worse and it becomes a vicious circle” (participant 26)*.

[38]

### 3.2. Multiple Factors Shaping Stigma

The second theme, multiple factors shaping stigma, has four sub-themes: ignorance and misunderstanding, spiritual and religious beliefs, family and friends and cultural beliefs (Figure 2).

#### 3.2.1. Ignorance and Misunderstanding

Stigma is often equated with ignorance or misunderstanding of mental health conditions [26] and there is a widespread misunderstanding and lack of awareness of mental health difficulties by people very close to those with mental illness [30]. For example, it is believed in some communities that a person could “catch” or be infected by psychosis [37]. Similarly, some people have rigid and pre-defined stereotypical beliefs about what depression looks like such as constant sadness or suicidality, which oversimplifies and misrepresents the condition [30]. Adolescents’ experiences of not being understood frequently co-occur with stigmatisation but extend beyond traditional definitions of stigma and constitute a distinct relational challenge [27].

Some people recognised that other people cared about them but lacked adequate understanding of their mental illness, leading many to consciously withdraw from these relationships to protect themselves from further emotional harm [30]. Misunderstanding from others triggered feelings of shame and guilt with damaging effect on relationships and everyday social interactions [30]. Fear of being misunderstood is as influential as fear of stigma in shaping adolescents’ disclosure decisions and a sense of not being understood discourages them from engaging in open disclosure about psychosis [27]. Some adolescents are willing to share their mental illnesses, if others show positive understanding [31] but anticipated misunderstanding can prevent them from disclosing or limiting disclosure [27]. Persistent lack of understanding leads to relational distance and emotional strain following disclosure [27]. People were asked to deny or dismiss their symptoms, showing a lack of understanding [33]. Adults may also respond to disclosure with dismissiveness or misunderstanding, undermining adolescents’ sense of being heard [27]. On the other hand, receiving genuine understanding, care, and support strengthened relationship bonds, improved social interactions, and contributed positively to their self-worth and overall wellbeing [30].

*“What I notice is that it is not well known. I find that people are not well informed, so there is a lot of confusion” (female, 35–45)*.

[24]

*“If you use the word, mental illness, nobody wants to go near that person because they don’t understand it”*.

[28]

#### 3.2.2. Spiritual and Religious Beliefs

Religious beliefs make it harder for people to accept mental illness, especially if faith is very important in their culture [35]. Spiritual explanations of mental illness contribute to stigmatisation such that mental illness is frequently understood to result from demonic involvement or spiritual attacks, as a punishment from spiritual entities or something caused by magic or the evil eye or by not praying or worshipping properly [28,35]. These spiritual interpretations are commonly reinforced by Christian clergy, particularly within Pentecostal settings, church members, relatives and peers [28]. Beliefs that mental illness results from sin or spiritual attack commonly prompted people to prioritise church-based spiritual interventions such as prayers, spiritual healing, and religious scripture over medical care [28,35].

*“The doctors couldn’t diagnose the illness. So, we perceived it was a spiritual illness. For so many months, I couldn’t eat and I grew very lean. So, my relatives referred to me as ‘the living dead”*.

[28]

*“People blame you for feeling like this and assume it’s from how distant you are from God and ask you to pray and read Quran [Muslim holy scripture]” (P8)*.

[33]

#### 3.2.3. Family and Friends

Individuals with mental illness often expected support from close family and friends [35,38]. However, for the majority of people, family and friends were unhelpful due to lack of understanding and awareness of mental health illness which led to negative emotional experiences for them [33]. For others, they receive minimal support from their families because of stigma and shame [36]. Families and friends were unhelpful in many ways: they dismissed or denied the illness, they did not realise the severity of mental illness, they ignored the individual, they compared people with mental health illness with themselves or with others, they asked them to pray more or rely on their faith to deal with mental illness, and they gave unsolicited or unhelpful advice and scolded them as lazy”, “uninspired”, or “weak” [31,33,35]. Some family members wanted to manage mental illness themselves, as they did not want their loved ones to be recorded in the mental illness registry because of an underlying distrust of Western medicine which some believe make symptoms worsen [33]. For some, stigma from immediate family members severely impacted on the self-esteem of individuals and led to crisis including self-harm and hospitalisation [33,38]. On the other hand, when family and friends understood and accepted mental illness, they reacted positively, provided support, and supported help-seeking which helped their loved ones to manage their illness and overcome challenges and protected them from stigma [35,38].

*“It’s mostly my mum, my cousins that are coming up against me... Like, instead of being there for me, instead of my cousin to be there for me, they were just saying, “Look, you are mentally ill, you are mentally ill”. “Go, come here, you are ill, you are ill.” That’s not how you should treat people. You shouldn’t be telling us, “You are mentally ill, you are mentally ill”*.

[28]

*“They (family members) do not talk to me. They do not support me. Sometimes, my parents are ashamed… My father is not proud of me in front of other people. For example, “my son is like this.” “See! My son has been like this.” What can he tell others? Other parents will say “my child goes to the college in Jayabaya (a university) takes informatics engineering field.” My parents do not mention about me like that. My father and mother do not do that…They are ashamed” (participant 10)*.

[36]

#### 3.2.4. Cultural Beliefs

Stigma towards mental illnesses is culturally specific and the role of stigma in mental health varies across different cultures [33]. Cultural beliefs contributed to stigma by framing mental illness as a permanent condition, even after apparent recovery [28]. Some cultures and communities do not understand mental health and were rejecting those with mental health problems because they do not exist as a concept in their culture [38]. As stigma and discrimination had impacted on sense of self and identity [38], some people considered giving up their entire cultural identity to prevent stigma [37].

*“Culture has influenced me in a way where I just don’t want to deal with it. Like knowing that it’s a negative thing, the more I’m like “Nah, I don’t want to deal with this right now” (L08/22/F/Indian)*.

[33]

*“In my culture mental health is not seen as mental health, mental health is seen as a sign of weakness. If you are stronger than you have stronger faith: especially with men you are supposed to take your problems and do with them and not let them get to you so that is why you know mental health is seeing very differently” (participant 26)*.

[38]

### 3.3. Management of Stigma

The third theme, management of stigma, has six sub-themes: management strategies, internalising stigma, isolation due to stigma, disclosure of mental illness, non-disclosure of mental illness and help-seeking for mental illness (Figure 2).

#### 3.3.1. Management Strategies

People with mental illness have their own strategies to manage stigma which are connected to handling the reactions of others and not merely the disease [35]. Coping strategies can be positive or negative [35], context-dependent [26] and may not always work as intended [27]. Positive strategies include talking with a therapist, having trusted and supportive relationships, focusing on positive things, accessing religious support, and using distraction techniques such as exercising, reading, and researching to understand the illness [26,35,37,38]. On the other hand, negative strategies include isolation and self-harm [25,26,35].

*“I personally cope by staying busy, so I have a proper routine in university. So, I gym, I go to the library, I study, I work, um, I try to be with friends, or I just try to keep myself as busy as I can so that—I’m not—I don’t have time to think about my um, problems” (Rafia, 22, female)*.

[26]

*“I always tried to isolate myself from people so no one would know the symptoms that I had or I’d lose someone” (P1)*.

[33]

#### 3.3.2. Internalising Stigma

Internalising stigma and prejudice leads individuals to accept negative stereotypes about themselves, doubt the legitimacy of their experiences, believe the stigma as a fact, and assume personal fault for problems which leads to frustration and personal, relational and psychological difficulties [23,31,32]. Some narratives are used to avoid or reduce shame and stigma, and these include an idealised past or childhood, emphasis on a previously normal life before the onset of mental distress and future planning as a strategy to maintain normality [25].

*“I just always think I’m making things up” and “I think everything is my fault” (Maggie, 19, female)*.

[13]

*“Someone told me, ‘It’s all in your head, you’re going crazy’- I believed it.” (Taylor, 23, female)*.

[13]

#### 3.3.3. Isolation Due to Stigma

Intentional isolation, social disengagement or reduced social interaction were used as coping strategies to manage stigma [30]. The reasons for these include anticipated stigma, fears of stigma, protection from the stigma, avoiding rejection or loss of status, preventing disclosure, feeling isolated and misunderstood, and breakdown of trust and relationship [29,30,37,38]. Although solitude protected people from stigma, it also increased the likelihood of dwelling on negative thoughts and emotions [30]. As a result, some people used social interactions either to present an enhanced version of themselves or to distract from their emotional difficulties [30]. For some people, neither social interaction nor solitude reliably improved their emotional state, leaving them feeling isolated regardless of the situation [30].

*“I prefer to spend time alone so that nobody will look down on me and nobody will stigmatize me. So, I prefer staying all alone…” (P13, 25, male)*.

[30]

*“It’s not like you weren’t friends with them but you just always kind of kept [teammates] at a distance” to protect against potential stigmatization and subsequent status loss”*.

[29]

#### 3.3.4. Disclosure of Mental Illness

Disclosure was used by some people to challenge stigma-driven silence, engage in public discussion, correct misrepresentations, combat or reduce public stigma and normalise mental illness [29,30,35]. Disclosing mental health issues to others resulted in healing, reassurance, a feeling of comfort, relaxation and freedom, deepening of relationships, and building of trust [31]. Others believe that disclosure will lead to awareness and understanding about mental illness that will bring reassurance and practical support [23,27].

*“Talking about mental health is crucial in helping to share experiences and normalise feelings. I would just talk about what happened to me, and for me that makes me feel better” (participant 4)*.

[23]

For some people, disclosure of mental illnesses caused unsupportive behaviours and stigmatising attitudes among loved ones [31]. Disclosing mental illnesses raised questions to others who needed further explanation [31] while some individuals felt uncomfortable being questioned by others about their mental illnesses and they were reluctant to share more information [31].

Disclosure can be both potentially supportive and socially risky [27] yet most people are advised to consider disclosure [31]. As a result, some people selectively disclosed their mental health diagnosis to people they trust, particularly to those who had similar lived experiences of depression or in circumstances when they felt safer [27,31]. Selective and limited disclosure of mental health experiences is a key strategy used to reduce anticipated stigma [27,31]. Decisions about disclosure involve careful judgement about the content, recipient, timing and method of sharing mental health information [27]. It reflects the combined influence of personal stigma beliefs, relationship factors and past experiences of both disclosure and concealment and wider social and cultural expectations [27].

*“... You sort of have to pick and choose, I suppose, who you talk to about it, depending on how you think they’ll react and what they’ll say. You’re more likely to share if you think they’re going to be like accepting, and not judging, and being sort of willing to listen and learn. Um. If you think you’re sort of going to get a bad reaction off them and they’ll think of you differently, or you see they are uncomfortable... you’re less likely” (P13, 18, female)*.

[31]

#### 3.3.5. Non-Disclosure of Mental Illness

Some people used non-disclosure methods such as lying, concealment, and secrecy, as well as hiding medication as a common strategy, to divert attention away from their mental health difficulties and avoid stigma altogether [26,28,30,37,38]. These people choose non-disclosure to feel in control and comfortable, avoid misunderstanding, gain more social acceptance, avoid shame and to be safe and secure [25,31,37]. Others choose non-disclosure due to previous stigma experience [29,31]. Some people stated that mental illness is a personal and private issue which is embarrassing to talk about [35]. Those who had visible signs of their mental health difficulties found secrecy hard to maintain and often struggled with the pressure to hide or explain these physical indicators [30].

*“It’s not everything that you have to discuss with people. When you tell them you were admitted at the psychiatric hospital, they will tell you have gone mad”*.

[28]

Some people stated that hiding their mental health condition meant keeping a large part of their lives from others, which left them feeling not fully known or understood [31]. Withholding personal information may be interpreted by others as dishonesty or a relational breach [27] while concealment of mental illness can lead to strained family interactions, breakdown of long-term intimate relationships and feelings of disconnection from wider social networks [29,31]. Secrecy played an unfavourable role in individuals’ recovery as they, for example, looked for more help-seeking from others while hiding real life experiences, which would create a vicious cycle [31]. Although secrecy helps young people to protect their self-esteem, it also creates more opportunities for stigma [31].

*“You don’t want to share anything because you even in turn realize these ideas that there will be negative effects if you do or people will view you in these ways if you do and... Yeah, yeah. You want to control how you portray yourself so that others can see you in a way that is not only your depression” (P27, 20, male)*.

[31]

#### 3.3.6. Help-Seeking for Mental Illness

Stigma towards mental illnesses leads to a lack of help-seeking behaviour or reluctance to seek help from mental health services [23,33,38]. A supportive environment and willingness to talk are essential for encouraging help-seeking behaviour [23]. Adolescents vary in how they interpret illness-related differential treatment, with some perceiving it as stigmatising and others experiencing it as supportive or accommodating [27]. Past experiences of stigma and discrimination within mental health services hindered engagement and most people reported they had not received good quality person-centred care [38]. People with mental illness noted that stigma was frequently manifested in the use of restraint or seclusion by healthcare teams [36]. The most frequent and severe forms of stigma were reported among those who had not been offered or had not received psychological input but those who accessed psychological support found it helpful for understanding themselves and for recognising positive aspects of themselves [37].

*“I was embarrassed to go and see a counsellor” (Jane, 22, female)*.

[13]

*“To treat me like an animal. They immediately picked me up and threw me on the bed and squeezed me hard and gave me an injection. That was back in the day. I don’t think people do that kind of thing anymore, but it was a very brutal hospitalization. And I wasn’t violent; I hadn’t done anything. I was talking a little too fast, I had a lot of energy but I wasn’t suicidal or anything” (male, 45–55)*.

[24]

It is important to note that some of the sub-themes under this theme relate to one another. Although all the sub-themes function primarily as strategies for managing stigma, and some of the sub-themes have secondary functions such as expressions and consequences. For example, isolation can be used to express stigma and can also be a consequence of stigma. Disclosure can be used to challenge stigma while non-disclosure can be an expression of stigma.

## 4. Discussion

This systematic review and meta-synthesis has provided an updated synthesis of qualitative evidence capturing the lived experience of stigma among people diagnosed with mental illness. Key findings fall into three main themes: the manifestation of stigma, multiple factors shaping stigma and the management of stigma. Beyond these themes, the findings can be further understood through an interpretive synthesis that explains how stigma operates across levels and over time. Stigma in the included studies is best understood not as a set of discrete categories but as a dynamic process that moves across interconnected levels including the self, family, healthcare and wider sociocultural contexts. At the societal and cultural level, stigma is generated through shared beliefs, media narratives and spiritual or moral interpretations that frame mental illness as deviant or weak [24,28,33]. These meanings are transmitted into family and community relationships where misunderstanding, shame and judgement reinforce stigma through everyday interactions such as dismissal, labelling and exclusion [33,35,38]. Within healthcare, these assumptions may become institutionalised, further legitimising stigma [36]. Across these domains, individuals internalise these messages, resulting in self-stigma characterised by shame, reduced self-worth and altered identity [23,32]. Stigma therefore circulates between structures, relationships and the self, with each level reinforcing the others over time [27,28,38].

Disclosure, concealment and help-seeking operate as interconnected processes through which individuals actively manage stigma. Disclosure involves a negotiated assessment of risk shaped by anticipated and prior stigma within specific relational and cultural contexts [27,31]. While it can challenge stigma, foster understanding and strengthen relationships, it also risks rejection and further stigmatisation, making it both enabling and threatening [27,31,35]. Concealment functions as a protective strategy that maintains social acceptance and control over identity but may reinforce isolation and disconnection over time [29,30,37]. These strategies directly shape help-seeking, as fear of stigma discourages service use while supportive environments facilitate it [23,33,38]. Together, they form a feedback loop in which stigma shapes behaviour and behaviour in turn reproduces stigma and limits recovery [27,31,38].

The findings also suggest that stigma is sustained through reinforcing feedback loops linking structural conditions, interpersonal interactions and individual responses. Cultural norms, religious interpretations and limited mental health literacy shape how stigma is expressed in families, communities and healthcare systems [28,33], influencing experiences of judgement and exclusion [35,38]. These interactions contribute to internalised stigma, shaping expectations of rejection and influencing behaviours such as withdrawal, selective disclosure or avoidance of services [29,30,32]. While protective in the short term, these responses reduce opportunities for supportive experiences and maintain social distance, thereby reinforcing stigma over time [31,38]. Stigma therefore operates as a self-sustaining system in which individual actions and social structures continually interact [27,28,38].

Taken together, this synthesis conceptualises stigma as a relational and process-driven phenomenon rather than a fixed attribute, showing how it is co-produced through interactions between beliefs, relationships and practices and actively managed in everyday life [27,28,35]. This highlights that interventions must move beyond single-level approaches to target the links through which stigma is transmitted, internalised and enacted [23,38]. Efforts that improve understanding, support safer disclosure and strengthen trusting relationships may be more effective than those focused only on attitudes or knowledge [23,38]. This provides a more integrated theoretical account of stigma as a dynamic system and a clearer basis for multi-level and context-sensitive responses [23,38].

A further implication is that stigma management involves ongoing identity work in which individuals continuously negotiate how they are seen by others and how they see themselves. Individuals construct and present versions of themselves that resist or adapt to stigmatising narratives, such as emphasising normality, distancing from more stigmatised diagnoses or selectively sharing information to maintain valued roles [25,27,34]. These practices are shaped by cultural expectations, family responses and anticipated reactions [33,38]. While they can preserve dignity and belonging, they also require sustained effort and reflect the constraints imposed by stigma. This shows that stigma not only limits help-seeking and inclusion but also reshapes identity and self-presentation over time [23,25,32].

Stigma is temporally dynamic, unfolding across stages of illness, diagnosis and recovery rather than occurring as a single event [26,27]. Early experiences of misunderstanding or labelling shape expectations of rejection, while repeated interactions with family, community and services reinforce or challenge these patterns [26,27,30,38]. Stigma is therefore cumulative and path-dependent, with past experiences influencing future decisions about disclosure, concealment and help-seeking [27,31]. This highlights the need for sustained, context-sensitive interventions delivered at multiple points, recognising stigma as an evolving process embedded within lived experience [28,38].

This review has demonstrated that mental illness is widely perceived as highly stigmatised, fuelled by stereotypes and negative societal views [26,31]. Stigma is pervasive and persistent, affecting people across all areas of life despite growing public discussion [13,24,28]. Kågström et al. [14] agree, suggesting that despite increased awareness campaigns there is persistent global mental health stigma with limited improvement to attitudes. Young people report that stigma remains widespread and personally impactful, leading some individuals to hide their mental health struggles [26,30]. Crockett et al. [15] also found that stigma is a barrier for young people accessing support for their mental health. Media and celebrity coverage attempt to reshape public perceptions of mental illness but often still misrepresent conditions [24]. Negative or sensationalised portrayals contribute to mixed messages and reinforce fear-based stereotypes [24,32]. Film and television frequently depict people with severe mental illnesses such as schizophrenia as dangerous or violent, influencing public attitudes [32]. Zhang & Firdaus [39] suggest that the media plays a significant role in shaping public perception of mental illness, and that negative portrayals reinforce stigma and public misunderstanding.

This review has identified three main forms of stigma that affect people with mental illness: self-stigma, public stigma and professional stigma; leading individuals to feel “not normal” and to encounter negative attitudes from healthcare professionals [23,32,36]. Families and friends may react with shock, denial or anger when a loved one receives a diagnosis, reflecting the influence of public stigma on close relationships [35]. These three forms of stigma are well-documented in the literature. For example, self-stigma has been shown to erode self-esteem and reinforce feelings of abnormality among people with mental illness [40]. Professional stigma also remains prevalent, with healthcare professionals frequently exhibiting negative or dismissive attitudes toward service users [41,42]. Public stigma continues to shape interpersonal dynamics, with systematic reviews highlighting how societal prejudice influences family and friends’ reactions including shock, denial, and anger when a loved one receives a mental health diagnosis [14,43].

As described in this review, stigma influences interpersonal, educational, occupational, and community experiences daily [28]. It operates both directly (negative comments) and indirectly (subtle behaviours, media narratives) [13]. People with mental illness are labelled as “crazy”, “attention-seeking”, or incapable [13,26,36]. Other studies have also confirmed that people with mental illness are routinely labelled with derogatory stereotypes, which contribute to social distancing, discrimination, and reduced participation in key life domains [44,45]. Diagnosis can provide validation, insight, and relief, but for others it reinforces inadequacy and reduced self-worth [26,30]. Young people report similar stigma regardless of whether they have a formal diagnosis [30]. Psychosis is heavily stigmatised, linked with labels such as “dangerous”, causing young people to manage their behaviour to avoid anticipated stigma [27,38]. For some, diagnosis can exacerbate feelings of inadequacy and reduced self-worth which is consistent with recent studies showing that internalised stigma is closely linked to diminished self-esteem and quality of life [14,40,46].

This review has shown that mothers with mental illness face labels such as “bad mother”, “weak woman” and “crazy” and work to present themselves as capable and responsible [34]. Some mothers strategically contrast their experiences with others to reduce blame and stigma [34]. Certain diagnoses such as depression are perceived as more understandable than psychosis, affecting how others respond [27]. Recently, Kent and Jeganathan [47] found that fear of judgement and stigma prevented mothers from accessing mental health support. Stigma harms self-esteem, relationships, social life, education, work, physical health and overall wellbeing [30,35]. It leads to shame, embarrassment, self-criticism, emotional distress, and reduced future opportunities [32,33,37]. Stigma can worsen symptoms, creating cycles of anxiety, paranoia, and heightened distress [38]. These findings are consistent with recent evidence demonstrating that stigma undermines psychosocial, educational, and occupational functioning, while also contributing to poorer physical and mental health outcomes [14,15].

As shown by this review, lack of understanding leads to withdrawal, emotional harm, and fear of being misunderstood [27,30]. Misunderstanding includes beliefs that mental illness is “infectious” or that depression must look a certain way [30,37]; however, genuine understanding strengthens relationships and improves wellbeing [30]. These findings echo previous evidence showing that poor mental health literacy sustains inaccurate symptom stereotypes and entrenched myths, further amplifying stigma [48,49,50,51].

It is clear from this review that some religious communities view mental illness as a spiritual attack, punishment, or demonic influence [28,35]. These beliefs often prioritise prayer or spiritual interventions over medical treatment [28]. Faith-based interpretations can worsen shame and increase pressure to engage in religious practice [33]. Recent evidence further shows that negative religious coping and coercive spiritual expectations can heighten shame and increase pressure to engage in religious practices, compounding emotional distress [52,53,54].

As shown in this review, families often minimise, dismiss, or misunderstand mental illness, leading to emotional harm and reduced support [33,36]. Stigma within the family can trigger crises, including self-harm and hospitalisation [38]. When families understand and accept mental illness, they encourage help-seeking and provide protective support [35,38]. Recent studies highlight how familial misunderstanding and stigma can exacerbate emotional harm, with family members’ negative assumptions and distancing behaviours significantly reducing perceived support [14,55,56].

This review has shown that cultural norms shape whether mental illness is seen as weakness, permanent, or shameful [28,33]. Some cultures do not recognise mental illness, leading to rejection or misunderstanding [38]. Cultural stigma can make individuals want to distance themselves from their cultural identity to avoid discrimination [37]. This is supported by recent evidence showing that in some cultural contexts, biomedical concepts of mental illness are absent or rejected, contributing to misunderstanding, dismissal, and rejection of those experiencing distress [52,53].

As shown in this review, people use a mix of positive (therapy, support, distraction, faith practices) and negative (isolation, self-harm) coping strategies to manage stigma [26,35,37,38]. These strategies focus not only on managing symptoms but also on handling others’ reactions [27,35]. The effectiveness of coping strategies varies depending on context and can sometimes backfire [26,27]. These patterns mirror findings from recent studies showing that individuals draw on a wide repertoire of coping responses from therapeutic and social strategies to withdrawal and avoidance when managing stigma [14,57,58]. Individuals adopt negative stereotypes, believing their symptoms are exaggerated or are their fault [13,23,31]. Internalised stigma fuels emotional, relational, and psychological difficulties [32]. People use narratives about past “normality” or future plans to reduce shame and maintain identity [25]. This reflects broader evidence showing that internalised stigma forms through endorsement of negative societal stereotypes, leading individuals to interpret their symptoms as personal failings or exaggerations [59,60]. Some people isolate themselves to avoid judgement, rejection, or anticipated stigma [29,30,38]. Isolation protects from stigma but increases negative thinking and emotional distress [30]. Some people engage socially but present an enhanced or “safer” version of themselves to reduce perceived risk [30]. This aligns with a recent study showing that many people maintain social connections by masking distress or modifying their self-presentation to reduce the perceived risk of stigma [59,60].

This review has shown that disclosure can challenge stigma, normalise mental illness, and improve relationships by building trust [29,31,35]. Sharing experiences can offer comfort, relief, and reassurance [23]. People carefully decide what to disclose, to whom, and when based on trust, past experiences, and cultural expectations [27,31]. Recent evidence similarly shows that thoughtful disclosure can reduce stigma, enhance mutual understanding, and strengthen interpersonal trust [61,62,63]. People hide symptoms or diagnoses (lying, secrecy, hiding medication) to avoid stigma, misunderstanding, or identity threats [26,30,37]. Non-disclosure can protect self-esteem but can also strain relationships and prevent genuine connection [27,29,31]. Concealment may hinder recovery and create cycles of shame and reduced help-seeking [31]. Recent studies further highlight that concealment can restrict access to support, reinforce shame, and reduce help-seeking, ultimately hindering recovery [62,64].

Based on this review, it is clear that stigma reduces engagement with mental health services, driving reluctance to seek support [23,33,38]. Also, previous negative treatment including restraint discourages future help-seeking [24,36] but supportive relationships and open communication make help-seeking more likely and beneficial [23,37]. All of this reflect recent evidence showing that stigma substantially reduces help-seeking, with individuals perceiving high stigma being markedly less likely to pursue care [14,15,65].

A key contribution of this review is the identification of concrete, practice-relevant mechanisms through which stigma is experienced and can be addressed within contemporary mental healthcare contexts [13,24,28]. The findings demonstrate that stigma is embedded in everyday interpersonal interactions, particularly through subtle forms of invalidation, dismissive language and anticipatory judgement, which influence help-seeking, disclosure and engagement with services [13,27,38]. This suggests that stigma reduction requires attention at the level of public awareness, and within routine clinical and social encounters [14,15,65]. In practice, this includes strengthening relational and communication skills among healthcare professionals, such as actively validating service users’ experiences, avoiding minimising responses and recognising the impact of micro-level stigma [41,42,44].

Structured approaches to support disclosure decision-making, including selective and safe disclosure, may also be beneficial, given that disclosure is shown to be an ongoing and context-dependent process rather than a single event [27,31]. Furthermore, the review highlights the importance of addressing stigma within wider relational and cultural systems, as family responses, cultural norms and spiritual interpretations play a significant role in shaping how stigma is experienced and managed [28,33,35]. This supports the need for culturally sensitive, family-inclusive psychoeducation and engagement with community and faith-based networks to improve understanding and support [52,53,55]. The findings indicate that some commonly used coping strategies, such as concealment and social withdrawal, may be protective in the short term but detrimental over time, suggesting that practitioners should adopt a more nuanced, person-centred approach that supports adaptive coping while addressing internalised stigma [30,31,38]. Collectively, these findings provide insight into how stigma can be recognised and addressed across clinical, community and cultural contexts, offering practical direction for improving engagement, recovery and wellbeing in people living with mental illness [14,58,64].

## 5. Strengths and Limitations

To the authors’ knowledge, this systematic review is the most recent review that examines stigma experienced by people diagnosed with mental illnesses. The selected studies in the last five years have provided the most recent insights into the stigma related to mental illnesses. This review followed a rigorous methodology, including PROSPERO registration, adherence to PRISMA guidelines [16], use of the Critical Appraisal Skills Programme [21] and ROBIS assessment [22], to ensure transparency, reliability and high quality. A major strength of this review is that the selected studies have been conducted in different countries, and this provides insights into how different cultural beliefs shape stigma towards mental health across the world.

This systematic review has some limitations that should be considered when interpreting the findings. The review is limited only to studies published in the English language potentially limiting the cultural diversity and global representativeness of the findings. While focus groups could have provided valuable understanding of shared norms, social interactions, and collective constructions of stigma, individual interviews were prioritised to explore personal lived experiences in depth. The review was limited to recent studies published between 2021 and 2025 to ensure contemporary relevance. A limited time frame enables a more recent, focused and concentrated discussion and tailored recommendations. However, this narrow publication window may have excluded earlier high-quality studies and longitudinal perspectives, thereby limiting the ability to assess changes in stigma experiences over time. There is a risk of publication bias, as unpublished studies and the grey literature were not included; however, included studies have been limited to peer-reviewed studies to ensure that a high level of rigour and quality is maintained.

### Recommendations

Based on the findings of this review, the following recommendations are proposed:

- Reducing mental health-related stigma requires coordinated, culturally sensitive interventions at multiple levels. Public education campaigns should improve mental health literacy and change misconceptions among the public.
- Healthcare systems should promote person-centred, non-judgemental care through professional training.
- Community-based programmes should engage families, religious leaders, and local stakeholders to foster supportive environments and safe disclosure to integrate mental health awareness and anti-stigma initiatives.
- Policy making should ensure equitable access to mental health services and protect the rights of mentally ill individuals, while continuous research is needed to evaluate interventions and adapt strategies to diverse cultural contexts.

## 6. Conclusions

This review highlights the widespread and complex nature of stigma experienced by individuals living with mental illnesses. The review shows that stigma exists in many aspects of everyday life, including interpersonal relationships, cultural beliefs and institutional settings. Stigma towards mental illnesses often appears in the form of negative labelling, social exclusion, misunderstanding and discrimination. Stigma is experienced both externally through public and professional attitudes and internally through self-stigma. Many individuals avoid or delay accessing professional support due to fear of being judged, past negative experiences, pressure from family and cultural expectations. This could create a vicious cycle in which stigma worsens the mental health of individuals and increases feelings of isolation, making recovery more challenging. Lack of knowledge and misunderstanding about mental illnesses, spiritual or religious interpretations, family attitudes, and cultural beliefs all play important roles in stigma related to mental health. While some of these factors can reinforce stigma, supportive family and community environments can help individuals feel accepted and supported in their recovery. People living with mental illnesses adopt a range of strategies to cope with stigma. These include selectively disclosing their diagnosis, concealing their condition, withdrawing from social interactions, or seeking supportive relationships with trusted individuals. While some of these strategies can help individuals protect themselves, build resilience, and maintain their wellbeing, others may unintentionally contribute to further social isolation and limit opportunities for recovery. The findings of this review emphasise the need to address stigma through comprehensive and culturally sensitive approaches. Efforts to improve mental health literacy, foster supportive family and community environments, and promote person-centred care within health systems are essential. Reducing stigma is crucial not only for improving access to mental health services but also for promoting dignity, social inclusion and the overall wellbeing of people living with mental illnesses.

## Figures and Tables

**Table 1:** Inclusion and exclusion criteria for selecting studies.

**Category**	**Inclusion Criteria**	**Rationale**
Participants	Individuals with diagnosed mental health condition/diagnosis. Individuals with lived experience of mental health stigma.	To focus on stigma related to mental health.
Phenomenon of Interest	Mental health is the primary and only cause of stigma. Major focus on lived experience of mental health stigma.	To capture stigma related to mental health and no other things.
Study Design	Qualitative studies. Mixed-methods studies with a distinct qualitative component.	To obtain subjective experience of mental health stigma and first-hand information and personal experiences.
Data Collection	In-depth individual interviews (e.g., one-to-one, face-to-face, or telephone/online interviews). Individual lived experience captured/reported separately from other participants.	To get an in-depth understanding of the participants’ individual lived experiences.
**Category**	**Exclusion criteria**	**Rationale**
Participants	Individuals with dementia.	Participants may be unable to adequately describe lived experiences due to cognitive decline or memory loss.
Phenomenon of Interest	Stigma is not primarily related to mental health. Studies focusing primarily on COVID-19-related stigma.	This may miss the focus on stigma related to mental health.
Study Design	Studies without a qualitative component.	This may not capture individual experience or personal elements.
Data Collection	Open-ended questionnaires or focus group discussions.	This may limit expression of lived experience due to writing ability or style.

**Table 2:** Characteristics of the selected studies.

Authors and Country	Aim	Study Design	Major Findings	Limitations
Ansell et al., 2024 [13] (Australia)	To qualitatively describe stigma experiences of young people with anxiety and depression, focusing on how stigma is expressed.	Semi-structured interviews Thematic analysis Sample (*n* = 13) Gender: 4 M/8 F/1 non-binary Age: 16–24; mean = 20.23	Six themes: (1) stigma is omnipresent; (2) denied, minimised, and blamed; (3) seen as less than others; (4) social undesirability; (5) self-doubt and internalisation; (6) withdrawal. Stigma remains pervasive despite increased mental health awareness.	Limited cultural diversity; mostly urban participants.
Edwards & Kotera, 2021 [23] (UK)	To explore institutional negativity and stigma in the UK police force towards mental ill health.	Semi-structured interviews Thematic analysis Sample (*n* = 5) Gender: 4 M/1 F Age: 43–62; mean = 52	Four themes: (1) police culture; (2) stigma of mental health; (3) disclosing mental illness; (4) breaking down barriers. Police culture and stigma significantly hinder help-seeking and disclosure.	Only one female participant; limited demographic diversity.
Favre et al., 2023 [24] (Switzerland)	To explore how perceived public stigma is described by people living with bipolar disorder (BD) and examine links between perceived public stigma and perceived public exposure.	Face-to-face in-depth interviews Thematic analysis Sample (*n* = 22) Gender: 7 M/15 F Age: Range not reported; mean = 47.1	Five themes: (1) mechanism of public stigma; (2) features of BD; (3) reaction to stigma; (4) public exposure; (5) impact of exposure. Public exposure influences stigma but does not eliminate prejudice or discrimination.	Voluntary participation bias; underrepresentation of social media impact due to older sample.
Georgaca et al., 2022 [25] (Greece)	To explore internalised stigma and self-presentation strategies of persons with psychotic and psychiatric experiences.	Biographical interviews Sequential and reconstructive analysis Sample (*n* = 6) Gender: 3 M/3 F Age: 26–42; mean = 32.3	Four themes: (1) portraying a retrospective “normal” self; (2) reluctance to disclose or make sense of distress; (3) fending off prospects of making sense of distressing experiences; (4) projecting a socially desirable self through future plans. These strategies reflect shame management and avoidance of stigma.	Biographical interviews with a psychologist may have influenced disclosure; lack of therapeutic relationship may have restricted depth of narratives.
Ghani & Bano, 2025 [26] (Pakistan)	To explore perceptions of mental illness, stigma, and stigma management strategies among university students.	Semi-structured phenomenological interviews Reflexive thematic analysis Sample (*n* = 11) Gender: 2 M/7 F, 1 other, 1 non-binary Age: 18–24; mean not reported	Three themes: (1) nature and perception of mental Illness; (2) experience of mental illness stigma; (3) coping with mental illness and associated stigma. Gender norms and academic pressures were key determinants of distress.	Sample from one private university; limited male representation; self-reported diagnoses.
Klauber et al., 2024 [27] (Denmark)	To explore adolescents’ experiences of stigma related to early-onset psychosis and their strategies for managing stigma.	Semi-structured interviews Phenomenological thematic analysis Sample (*n* = 34) Gender: 11 M/23 F Age: 14–19; mean not reported	Six themes: (1) non-disclosure: strategic management of stigma; (2) disclosure of psychosis; (3) a complex of non-disclosure considerations; (4) non-disclosure: a trial-and-error approach; (5) stigma management as an ongoing process; (6) openness: both therapeutic disclosure and normative context. Fear and experience of not being understood emerged as a central theme influencing disclosure decisions.	Single-interview design limits longitudinal insight.
Mfoafo-M’Carthy & Grischow, 2022 [28] (Ghana)	To explore lived experiences of stigma and its impact on wellbeing among Ghanaians diagnosed with mental illness.	Semi-structured interviews Inductive thematic analysis Sample (*n* = 10) Gender: 4 M/6 F Age: 19–40; mean = 34	Five themes: (1) beliefs in spiritual causes of mental illness; (2) spirituality as coping mechanism; (3) mistreatment and abuse; (4) social exclusion; (5) stigma. Stigma drove fear, shame, and avoidance of treatment.	Sample from one psychiatric hospital; reliance on opportunistic sampling; cultural specificity.
Petersen et al., 2024 [29] (Canada/USA)	To explore how mental ill-health stigma in sport impacts athletes’ social relationships and interactions.	Semi-structured interviews Reflexive thematic analysis Sample (*n* = 12) Gender: 5 M/7 F Age: 23–28; mean = 24.8	Two themes: (1) relational drawbacks to protecting oneself from stigmatisation; (2) growth through stigmatised experiences. Participants often withdrew socially and struggled to maintain relationships, yet supportive and open environments fostered meaningful connections and personal growth.	Snowball sampling may bias toward similar experiences; did not analyse differences by gender or sport.
Prizeman et al., 2023 [30] (UK)	To explore how public and internalised stigma related to depression influences loneliness, social isolation, and relationship quality among young people.	Semi-structured interviews Thematic analysis Sample (*n* = 22) Gender: 9 M/12 F/other 1 Age: 17–25; mean = 22	Four themes: (1) others’ misunderstanding of mental health disorders and the impact misunderstanding has on relationships; (2) effects of stigma on self and wellbeing; (3) stigma fosters secrecy vs. disclosure; (4) stigma increases loneliness via avoidance of social contexts. Stigma around depression makes young people feel misunderstood and judged, leading to secrecy, isolation, and weaker relationships.	Cannot fully disentangle stigma effects from depression symptoms.
Prizeman et al., 2024 [31] (UK)	To explore how stigma influences secrecy and disclosure of depression among young people and its impact on loneliness and social isolation.	Semi-structured interviews using conceptual model drawings Thematic analysis Sample (*n* = 28) Gender: 8 M/19 F/other 1 Age: 18–25; mean = 21.3	Four themes: (1) depression secrecy, positive and negative aspects; (2) depression disclosure, positive and negative aspects; (3) selective disclosure as a solution; (4) recommendations vs. personal preferences. Stigma around depression pushes young people toward secrecy and cautious sharing, which often worsens loneliness and isolation.	Possible self-selection bias; did not include severely depressed individuals.
Rodwin et al., 2023 [32] (USA)	To examine stigma experiences and factors associated with anticipated stigma among historically marginalised young adults with serious mental illness (SMI).	Semi-structured interviews Multivariable regression and grounded theory coding Sample (*n* = 57) Gender: (only available for the quantitative sample *n* = 113) 78 M/35 F Age: 18–34; mean = 26.4 years	Three themes—(1) perceptions of self; (2) societal views of people living with SMI; (3) impact of stigma on life: social, emotional, and behavioural. Young adults described stigma as shaping their self-perceptions, societal views, and negatively impacting their social, emotional, and behavioural lives.	Excluded nonbinary gender identities; qualitative protocol did not explicitly probe stigma; gender breakdown not available for qualitative sample.
Samari et al., 2022 [33] (Singapore)	To explore the role of perceived stigma from family and friends in help-seeking behaviours among young people with depression.	Semi-structured interviews Thematic analysis Sample (*n* = 33) Gender: 15 M/18 F Age: 20–35; mean = 26	Four themes: (1) absence of support; (2) provision of unhelpful support; (3) preference for non-disclosure; (4) opposition to formal help-seeking. Cultural factors (e.g., collectivism, religious beliefs) reinforced stigma and delayed professional help-seeking.	Did not examine differences by gender or relationship dynamics.
Schamschula & Paul, 2024 [34] (Austria)	To explore how mothers with a mental illness position themselves and how these reveal stigmatising discourses around motherhood and mental illness.	Semi-narrative interviews Thematic and rhetorical discourse analysis Sample (*n* = 20) Gender: 20 F Age: 24–46; mean = 37	Three themes: (1) deconstructing the narrative of a bad mother; (2) deconstructing the narrative of a not normal/crazy woman; (3) deconstructing the narrative of a weak person. Contrast devices allowed mothers to challenge stigma and maintain identity as ‘good mothers’ despite illness.	Findings context-dependent; analysis limited to contrast devices, other strategies not explored in depth.
Sharif et al., 2025 [35] (Saudi Arabia)	To explore experiences of stigma among people with mental health disorders in Saudi Arabia, and how cultural and religious factors influence stigma and coping strategies.	Qualitative descriptive design Reflexive thematic analysis Sample (*n* = 13) Gender: 1 M/12 F Age: 20–42; mean age not reported	Four themes: (1) effects on everyday life; (2) challenges; (3) overcoming challenges; (4) types of stigma. Participants emphasised need for awareness campaigns and culturally sensitive interventions.	Convenience sampling via social media; majority were students.
Subu et al., 2021 [36] (Indonesia)	To explore types of stigma experienced by patients with mental illness and mental health nurses in Indonesia.	Semi-structured interviews Deductive qualitative content analysis Sample (*n* = 30) Gender: 17 M/13 F Age: Patients: 21–52 years, nurses: 22–43 years; mean age not reported.	Five themes: (1) perceived stigma from a patient perspective; (2) public stigma; (3) family attitudes towards mentally ill patients; (4) employment discrimination; (5) professional stigma. Cultural and religious beliefs were found to reinforce stigma.	Participants recruited from a single hospital; The analysis relied heavily on researchers’ interpretation of latent meanings in interview data, which introduces subjectivity.
Vyas et al., 2021 [37] (UK)	To explore stigma experiences of second-generation British South-Asian people using Early Intervention in Psychosis (EIP) services.	Semi-structured interviews Thematic analysis Sample (*n* = 10) Gender: 8 M/2 F Age: 19–39; mean age not reported.	Four themes: (1) the burden of silencing; (2) unrecognised aspects of their situation by services; (3) experience as “the other”; (4) finding ways to cope. Intersectionality of mental health, race, religion, and class amplified distress caused by stigma.	Predominantly male participants; stigma may have deterred some from participating.
Wood et al., 2022 [38] (UK)	To explore experiences of stigma and discrimination among ethnic minority service users with psychosis.	Semi-structured interviews Thematic analysis Sample (*n* = 21) Gender: 16 M/5 F Age: 21–54; mean = 33.9	Five themes: (1) social and cultural context of stigma; (2) stigma is a family problem; (3) stigma and discrimination within mental health system; (4) intrapersonal impacts; (5) navigating interpersonal relationships is a challenge. Participants face widespread stigma across social, family, and healthcare settings, causing shame, isolation, and barriers to recovery, while coping often relies on concealment and personal strategies like prayer.	Focused broadly on ethnic minority groups, may miss nuances of specific cultures; relatively short interviews.

**Table 3:** Quality appraisal of the selected studies is based on CASP (2024) [21]. A score of 10 is for Yes, 5 is for can’t tell and 0 is for No. An overall score of 0–20% is very poor, 20–40% is poor, 40–60% is moderate, 60–80% is good and 80–100% is excellent.

Study	Q1. Was There a Clear Statement of the Aims of the Research?	Q2. Is a Qualitative Methodology Appropriate?	Q3. Was the Research Design Appropriate for the Aims of the Research?	Q4. Was the Recruitment Appropriate for the Aims of the Research?	Q5. Was the Data Collected in a Way That Addressed the Research?	Q6. Has the Relationship Between the Researcher and Participant Been Adequately Considered?	Q7. Have Ethical Issues Been Taken into Consideration?	Q8. Was the Data Analysis Sufficiently Rigorous?	Q9. Is There a Clear Statement of Findings?	Q10. How Valuable is the Research?	Score (%)
**Ansell et al., 2024** [13]	Yes	Yes	Yes	Yes	Yes	No	Yes	Yes	Yes	Yes	90
**Edwards and Kotera 2021** [23]	Yes	Yes	Yes	Yes	Yes	No	Can’t tell	Yes	Yes	Yes	85
**Favre et al. 2023** [24]	Yes	Yes	Yes	Yes	Yes	No	Yes	Yes	Yes	Yes	90
**Georgaca et al. 2022** [25]	Yes	Yes	Yes	Yes	Yes	No	Yes	Yes	Yes	Yes	90
**Ghani and Bano 2025** [26]	Yes	Yes	Yes	Yes	Yes	No	Yes	Yes	Yes	Yes	90
**Klauber et al. 2024** [27]	Yes	Yes	Yes	Yes	Yes	No	Yes	Yes	Yes	Yes	90
**Mfoafo-MGÇÖCarthy and Grischow 2022** [28]	Yes	Yes	Yes	Yes	Yes	No	Yes	Yes	Yes	Yes	90
**Petersen et al. 2024** [29]	Yes	Yes	Yes	Yes	Yes	No	Yes	Yes	Yes	Yes	90
**Prizeman et al. 2023** [30]	Yes	Yes	Yes	Yes	Yes	No	Yes	Yes	Yes	Yes	90
**Prizeman et al. 2024** [31]	Yes	Yes	Yes	Yes	Yes	No	Yes	Yes	Yes	Yes	90
**Rodwin et al. 2023** [32]	Yes	Yes	Yes	Yes	Yes	No	Yes	Yes	Yes	Yes	90
**Samari et al. 2022** [33]	Yes	Yes	Yes	Yes	Yes	No	Yes	Yes	Yes	Yes	90
**Schamschul and Paul 2024** [34]	Yes	Yes	Yes	Yes	Yes	No	Yes	Yes	Yes	Yes	90
**Sharif et al. 2025** [35]	Yes	Yes	Yes	Yes	Yes	No	Yes	Yes	Yes	Yes	90
**Subu et al. 2021** [36]	Yes	Yes	Yes	Yes	Yes	No	Yes	Yes	Yes	Yes	90
**Vyas et al. 2021** [37]	Yes	Yes	Yes	Yes	Yes	No	Yes	Yes	Yes	Yes	90
**Wood et al. 2022** [38]	Yes	Yes	Yes	Yes	Yes	No	Yes	Yes	Yes	Yes	90

**Table 4:** Risk of bias analyses for the review (ROBIS) tool (Whiting et al. 2016) [22].

**Phase 1: Assessing relevance**		
State your overview/guideline question (target question) and the question being addressed in the review being assessed:		
**Category**	**Target Question (e.g., Overview or Guideline)**	**Review Being Assessed**
Patients/Population(s):	Individuals with a lived experience of mental health (MH) stigma/discrimination of age categories of adolescents and adults)	Individuals who have been diagnosed with a mental health (MH) disorder
Exposure(s) and comparator(s):	Stigma related to mental health	Stigma related to mental health
Outcome(s):	Lived experience of stigma related to mental health	Lived experience of stigma related to mental health
Does the question addressed by the review match the target question?		Yes
**Phase 2: Identifying concerns with the review process**		
**DOMAIN 1: STUDY ELIGIBILITY CRITERIA**		
Describe the study eligibility criteria, any restrictions on eligibility and whether there was evidence that objectives and eligibility criteria were pre-specified:		
1.1 Did the review adhere to pre-defined objectives and eligibility criteria?		Yes
1.2 Were the eligibility criteria appropriate for the review question?		Yes
1.3 Were the eligibility criteria unambiguous?		Yes
1.4 Were any restrictions in the eligibility criteria based on study characteristics appropriate (e.g., date, sample size, study quality, outcomes measured)?		Yes
1.5 Were any restrictions in the eligibility criteria based on sources of information appropriate (e.g., publication status or format, language, availability of data)?		Yes
Concerns regarding specification of study eligibility criteria		Low
Rationale for concern		Eligibility criteria were clearly defined and aligned with the review question. However, restriction to English-language studies may have excluded relevant evidence.
**DOMAIN 2: IDENTIFICATION AND SELECTION OF STUDIES**		
Describe the methods of study identification and selection (e.g., number of reviewers involved):		
2.1 Did the search include an appropriate range of databases/electronic sources for published and unpublished reports?		Yes
2.2 Were methods additional to database searching used to identify relevant reports?		Yes
2.3 Were the terms and structure of the search strategy likely to retrieve as many eligible studies as possible?		Yes
2.4 Were restrictions based on date, publication format, or language appropriate?		Yes
2.5 Were efforts made to minimise error in selection of studies?		Yes
Concerns regarding methods used to identify and/or select studies		Low
Rationale for concern		The search included relevant databases and was systematically conducted; however, a review of the reference lists of the 17 identified studies yielded no new relevant studies. The grey literature was not searched as the focus of the review is on peer-reviewed academic journals.
**DOMAIN 3: DATA COLLECTION AND STUDY APPRAISAL**		
Describe methods of data collection, what data were extracted from studies or collected through other means, how risk of bias was assessed (e.g., number of reviewers involved) and the tool used to assess risk of bias:		
3.1 Were efforts made to minimise error in data collection?		Yes
3.2 Were sufficient study characteristics available for both review authors and readers to be able to interpret the results?		Yes
3.3 Were all relevant study results collected for use in the synthesis?		Yes
3.4 Was risk of bias (or methodological quality) formally assessed using appropriate criteria?		Yes
3.5 Were efforts made to minimise error in risk of bias assessment?		Yes
Concerns regarding methods used to collect data and appraise studies		Low to moderate
Rationale for concern		Data extraction and quality appraisal were conducted systematically. However, poor reflexivity in the selected studies may influence interpretation of findings.
**DOMAIN 4: SYNTHESIS AND FINDINGS**		
Describe synthesis methods:		
4.1 Did the synthesis include all studies that it should?		Yes
4.2 Were all pre-defined analyses reported or departures explained?		Yes
4.3 Was the synthesis appropriate given the nature and similarity in the research questions, study designs and outcomes across included studies?		Yes
4.4 Was between-study variation (heterogeneity) minimal or addressed in the synthesis?		Yes
4.5 Were the findings robust, e.g., as demonstrated through funnel plot or sensitivity analyses?		Not applicable
4.6 Were biases in primary studies minimal or addressed in the synthesis?		Yes
Concerns regarding the synthesis and findings		Low
Rationale for concern		Thematic analysis and synthesis were appropriate for qualitative evidence and the findings were robust. However, funnel plot or sensitivity analyses were not used.
**Phase 3: Judging risk of bias**		
Summarise the concerns identified during the Phase 2 assessment:		
**Domain**	**Concern**	**Rationale for Concern**
1. Concerns regarding specification of study eligibility criteria	Low	Eligibility criteria were clearly defined and aligned with the review question. However, restriction to English-language studies may have excluded relevant evidence.
2. Concerns regarding methods used to identify and/or select studies	Low	The search included relevant databases and was systematically conducted; however, a review of the reference lists of the identified 17 studies yielded no new relevant studies. The grey literature was not searched as the focus of the review is on peer-reviewed academic journals.
3. Concerns regarding methods used to collect data and appraise studies	Low to moderate	Data extraction and quality appraisal were conducted systematically. However, poor reflexivity in the selected studies may influence interpretation of findings.
4. Concerns regarding the synthesis and findings	Low	Thematic analysis and synthesis were appropriate for qualitative evidence and the findings were robust. However, funnel plot or sensitivity analyses were not used.
RISK OF BIAS IN THE REVIEW		
Describe whether conclusions were supported by the evidence:		
A. Did the interpretation of findings address all of the concerns identified in Domains 1 to 4?		Yes
B. Was the relevance of identified studies to the review’s research question appropriately considered?		Yes
C. Did the reviewers avoid emphasising results on the basis of their statistical significance?		Yes
Risk of bias in the review risk		Low to moderate
Rationale for risk		While rigorous methods were applied in study selection, appraisal, and synthesis, limited reporting of reflexivity in the selected studies should be considered when interpreting the findings.

## Data Availability

No new data were created or analyzed in this study. Data sharing is not applicable to this article.

## Supplementary Materials

The following supporting information can be downloaded at: https://www.mdpi.com/article/10.3390/ijerph23070873/s1. File S1: PRISMA 2020 Checklist.
